# Virtual monochromatic spectral CT imaging in preoperative evaluations for intraductal spread of breast cancer: comparison with conventional CT and MRI

**DOI:** 10.1007/s11604-023-01392-4

**Published:** 2023-02-02

**Authors:** Yuko Matsuura, Takeshi Kamitani, Koji Sagiyama, Yuzo Yamasaki, Takuya Hino, Makoto Kubo, Hideki Ijichi, Hidetaka Yamamoto, Hidetake Yabuuchi, Kousei Ishigami

**Affiliations:** 1 Department of Clinical Radiology, Graduate School of Medical Sciences, Kyushu University, 3-1-1, Maidashi, Higashi-Ku, Fukuoka, Japan; 2 Department of Surgery and Oncology, Graduate School of Medical Sciences, Kyushu University, 3-1-1 Maidashi, Higashi-Ku, Fukuoka, 812-8582 Japan; 3 Department of Surgery and Science, Graduate School of Medical Sciences, Kyushu University, 3-1-1 Maidashi, Higashi-Ku, Fukuoka, 812-8582 Japan; 4 Department of Anatomic Pathology, Graduate School of Medical Sciences, Kyushu University, 3-1-1 Maidashi, Higashi-Ku, Fukuoka, 812-8582 Japan; 5 Department of Health Sciences, Graduate School of Medical Sciences, Kyushu University, 3-1-1 Maidashi, Higashi-Ku, Fukuoka, 812-8582 Japan

**Keywords:** Breast cancer, Spectral CT, Virtual monochromatic image, Intraductal spread

## Abstract

**Purpose:**

To investigate the efficacy of virtual monochromatic spectral computed tomography imaging (VMI) in the preoperative evaluation for intraductal spread of breast cancer.

**Materials and methods:**

Twenty-four women who underwent spectral CT and were pathologically diagnosed with ductal carcinoma with a ≥ 2-cm noninvasive component were retrospectively enrolled in Group 1. Twenty-two women with 22 lesions pathologically diagnosed with ductal carcinoma in situ or microinvasive carcinoma were enrolled in Group 2. We compared the contrast-to-noise ratios (CNRs) of the lesions on conventional 120-kVp CT images and 40-keV VMIs in Group 1. Two board-certified radiologists measured the maximum diameters of enhancing areas on 120-kVp CT, 40-keV VMI, and MRI in Group 2 and compared with histopathological sizes.

**Results:**

The quantitative assessment of Group 1 revealed that the mean ± SD of the CNRs in the 40-keV images were significantly greater than those in the 120-kVp images (5.5 ± 1.9 vs. 3.6 ± 1.5, *p* < 0.0001). The quantitative assessment of Group 2 demonstrated that the lesion size observed in the conventional 120-kVp CT images by both readers was significantly underestimated as compared to the histopathological size (*p* = 0.017, 0.048), whereas both readers identified no significant differences between the lesion size measured on 40-keV VMI and the histopathological data. In a comparison with MRI, 40-keV VMI provided measurement within a 10-mm error range in more lesions as compared to the conventional 120-kVp CT.

**Conclusion:**

VMI improves the evaluation of intraductal spread and is useful for the preoperative evaluations of breast cancer.

## Background

Accurate measurements of a tumor size and extent are important in the selection of the surgical procedures to be conducted for breast cancer. An underestimation of ductal spread in a breast-conserving surgery can lead to a lower probability of curative resection, whereas an overestimation can lead to oversurgery. An accurate preoperative evaluation of intraductal spread is thus clinically important. In the assessments of the intraductal spread of breast cancer, mammography, ultrasound, and MRI have been reported to provide sensitivities of 21%–55%, 21%–89%, and 67%–93%, specificities of 86%–100%, 76%–86%, and 60%–90%, and accuracies of 42%–72%, 50%–85%, and 66%–92% [1–4]. Because MRI can generate images with higher contrast as compared to CT, MRI is considered the standard for the assessment of intramammary spread [5–7], but with its shorter acquisition time, CT can provide whole-body views for cancer staging [5–7]. In addition, the location of the lesions on MRI, which is usually performed with the patient in the prone position for the evaluation of the breast, is sometimes different from that identified in the patient’s surgery, which is performed with the patient in the supine position. The use of MRI can also be problematic for patients with claustrophobia or a metallic implant. CT has advantages such as the use of the same body position as the surgery, a short scan time, and the ability to detect metastases.

Spectral CT can generate virtual monochromatic imaging (VMI) by using the dual-energy technique. Low-energy-level VMI can increase the contrast enhancement of breast cancer, resulting in good contrast with soft tissue [8, 9]. We speculated that if the use of low-energy VMI could improve the detectability of intramammary spread, a comprehensive assessment could be performed without an MRI examination. We thus conducted the present study to investigate the efficacy of VMI in preoperative evaluations of the intraductal spread of breast cancer.

## Materials and methods

### Patients

This retrospective study was approved by the Ethical Review Board on Clinical Studies of our institution. The requirement of informed consent was waived. All CT and MR examinations were clinically indicated, having been requested by clinicians as part of the patients’ preoperative evaluation.

The Group 1 patients were retrospectively enrolled for the measurement of the contrast-to-noise ratio (CNR). The inclusion criteria for Group 1 were as follows; the patient (1) had undergone a spectral CT examination at our institution between May 2018 and February 2019, (2) had subsequently undergone surgical resection without neoadjuvant chemotherapy, and (3) was pathologically diagnosed with ductal carcinoma with a noninvasive component that was ≥ 2 cm, i.e., ductal carcinoma in situ (DCIS) ≥ 2 cm or invasive ductal carcinoma (IDC) with a ≥ 2-cm intraductal spread. These inclusion criteria were used based on our speculation that lesions with some degree of spread are suitable for CNR measurement.

The cases of the patients in Group 2 were collected for lesion size measurement. The inclusion criteria for Group 2 were as follows; the patient (1) had undergone both of spectral CT and MRI examinations at our institution between May 2018 and December 2021, (2) had subsequently undergone surgical resection without neoadjuvant chemotherapy, and (3) was pathologically diagnosed with DCIS or microinvasive carcinoma. We limited the patient population to those with DCIS or microinvasive carcinoma in order to evaluate only intraductal spread, because we speculated that an existence of invasive foci makes the recognition of the lesions be easier.

### CT protocols

All patients underwent chest-abdomen or chest-abdomen-pelvis CT in the supine position for the systemic staging of breast cancer. All volumetric data were acquired using dual-layer, dual-energy CT (IQon spectral CT, Philips Healthcare, Best, The Netherlands) after an intravenous injection of iodinated contrast media (Iopamidol, iohexol, or ioversol; 9.0 mgI/s/kg, delay time 80 s). The virtual 40-keV monochromatic images (40-keV VMIs) and the conventional 120-kVp CT images were reconstructed. The protocols were as follows: slice thickness, 0.675 mm; detector collimation, 0.675 × 64 mm; matrix size, 512 × 512; tube voltages, 120 kV; rotation time, 0.4 s/rotation; focus size, high; field of view (FOV), 400 mm; reconstructed thickness, 1 mm. The 40-keV VMIs were reconstructed with a spectral level of 0, and the conventional 120-kVp CT images were reconstructed with an iDose level of 4.

### MRI protocols

MR imaging was performed on a 3-Tesla system (Ingenia or Achieva, Philips Healthcare, Best, The Netherlands). The patient was in the prone position during the examination. A standard 16-channel sensitive encoding (SENSE) breast coil was used in all cases. For the dynamic MR imaging, gadopentetate dimeglumine (gadobutrol; 0.1 mmol/kg body weight [BW], 1 mL/sec) or meglumine gadoterate (0.1 mmol/kgBW, 2 mL/sec) was administered intravenously. Dynamic contrast-enhanced MR images were obtained using coronal T1 high-resolution isotropic volume excitation (THRIVE) (repetition time [TR], 3.8–3.9 ms; echo time [TE], 1.3 ms; flip angle, 15°; FOV, 32 × 32 cm; matrix, 320 × 317–330; slice thickness, 1 mm; number of excitations [NEX], 1; acquisition time, 90 s) before and at 0, 90, 180, 270 and 360 s after the administration of contrast medium. Axial high-resolution images were subsequently obtained using THRIVE (TR, 5.3 ms; TE, 2 ms; flip angle, 10°; FOV, 32 × 32 cm; matrix, 604 × 603; slice thickness, 0.3–0.9 mm; NEX, 1).

### Image analysis

#### Group 1: CNR

The CNR of the lesions was calculated as the difference between the mean CT value of the breast cancer lesion and the mean CT value of the surrounding breast tissue divided by the standard deviation (SD) of the CT value of the surrounding breast tissue. The CNR values of the conventional 120-kVp CT images and 40-keV VMI were measured by one board-certified radiologist (9 years of experience). For these measurements, free-hand regions of interest (ROIs) were manually placed on the slice in which the maximum diameter of the lesions was depicted using a commercially available viewer (SYNAPSE 5, FUJIFILM Medical, Tokyo, Japan).

#### Group 2: Lesion size measurement

The maximum diameters of enhancing areas were measured using multiplanar reconstruction (MPR) images on conventional 120-kVp CT images, 40-keV VMI, and MRI. Two board-certified radiologists (9 and 22 years of experience, respectively) evaluated each image independently on a viewer (SYNAPSE 5). They were aware that the subjects were DCIS or microinvasive carcinoma; however, they had no knowledge of the pathological sizes. No other imaging, such as mammography or ultrasonography, was referred. The CT and MRI reading were performed separately and the order of them was random for each case. The window levels and widths and the MPR cross-sectional direction were freely changed on the viewer by the observers. On MRI reading, they measured the lesion sizes on the images for which they deemed the contrast was the most favorable and on which the lesions were easy to recognize, referring to each phase of dynamic MRI and the subsequent axial high-resolution images.

### Histopathological assessment

Sectioning of resected specimens at 5-mm slices was performed according to the General Rules for Clinical and Pathological Recording of Breast Cancer (18th edition) established by the Japanese Breast Cancer Society. Pathologists mapped the tumor extension, and measured the invasive size, noninvasive size, and overall tumor diameters.

### Statistical analyses

In Group 1, the CNRs of the lesions were compared between the conventional 120-kVp CT images and the 40-keV VMIs by paired *t*-test. In Group 2, the lesion sizes measured on conventional 120-kVp CT images, 40-keV VMIs, and MRI were compared with the histopathological sizes by a one-way analysis of variance (ANOVA) followed by Dunnett's multiple comparison test. *P*-values < 0.05 were considered significant. The numbers of lesions within a 10-mm error range compared with the histopathological size or the MRI result were examined with the Bland–Altman test. We assessed the interobserver reliability of the conventional 120-kVp CT, the 40-keV VMI, and the MRI by determining the intraclass correlation coefficients (ICC). The statistical analyses were performed using GraphPad Prism9 (GraphPad Software, San Diego, CA, USA) or JMP® Pro 16.0.0 (SAS Institute Inc., Cary, NC, USA).

## Results

### Group 1: CNR

**Fig. 1:**
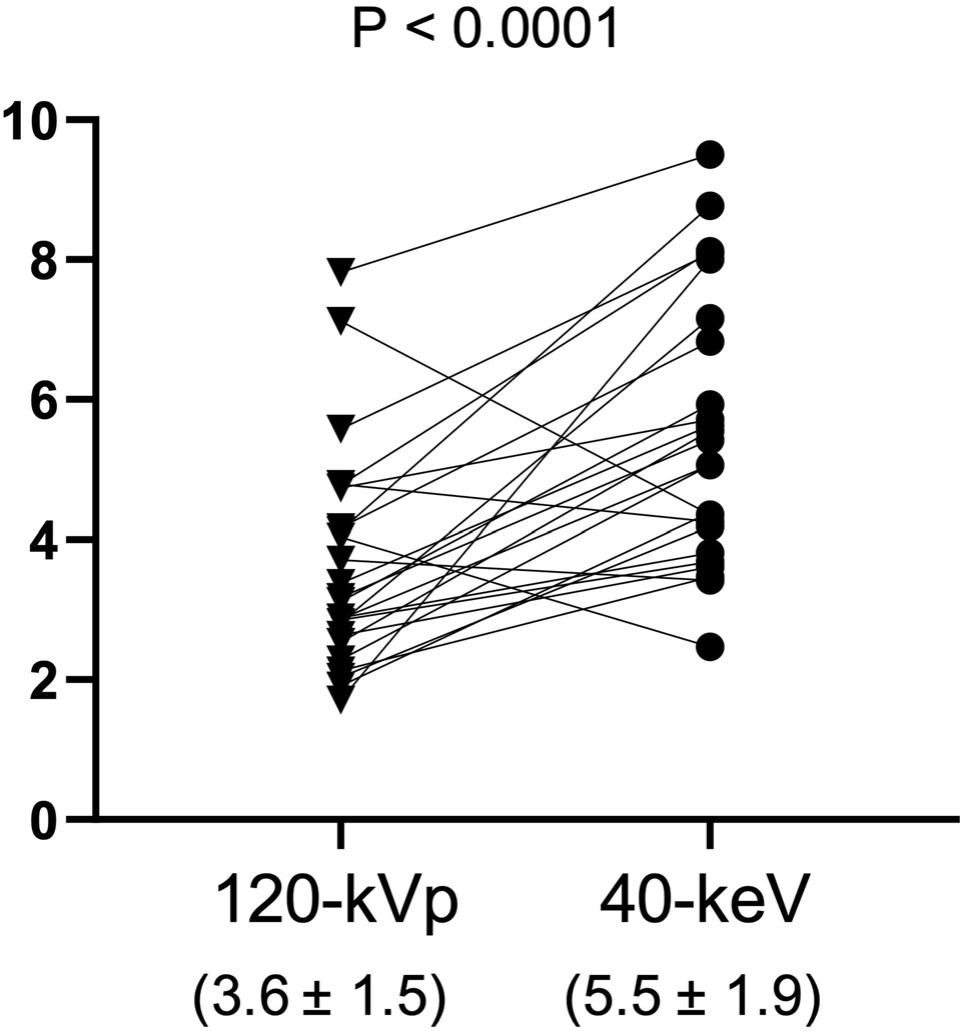
Comparison of the contrast-to-noise ratio (CNR) between 40-keV VMIs and conventional 120-kVp CT. The CNRs of the 40-keV VMIs results were significantly higher than those of the conventional 120-kVp CT images (*P* < 0.0001)

**Fig. 2:**
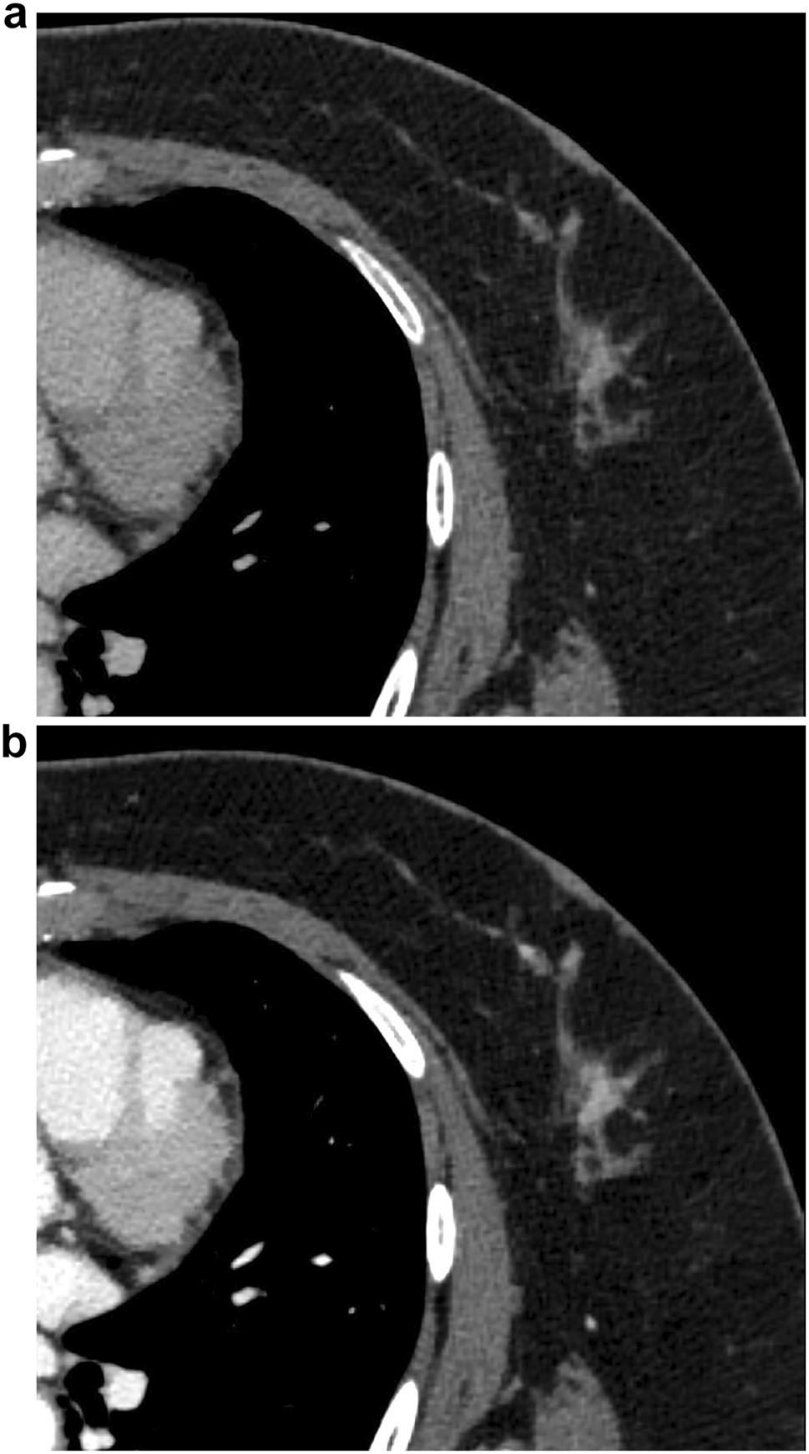
A 55-year-old female with microinvasive carcinoma (Group 1). The CNR was 2.13 on conventional 120-kVp (**a**) and 3.47 on 40-keV VMI (**b**). The use of 40-keV VMI improved the CNR

Twenty-four females were retrospectively enrolled in Group 1. The patients’ characteristics are shown in Table 1. The CT dose index-volume (CTDIvol) values for the spectral CT were 7.5–31.4 mGy (median 12.1 mGy) and the dose length product (DLP) values were 386.2–2225.0 mGy･cm (median 675.0 mGy･cm). The mean of the CNRs of the 40-keV images was significantly higher than that of the 120-kVp images (mean ± SD, 5.5 ± 1.9 vs. 3.6 ± 1.5, *p* < 0.0001) (Figs. 1, 2).

#### Group 2: lesion size measurement

Twenty-eight women who underwent both spectral CT and MRI at our institution between May 2018 and December 2021 and were pathologically diagnosed with DCIS or microinvasive carcinoma were retrospectively enrolled in Group 2. We excluded four cases without 1-mm slice images of 40-keV VMI, one case with a lesion that was difficult to identify on 40-keV VMI, and another case with a lesion that was difficult to identify on MRI. The remaining 22 women were assigned to Group 2. The patients’ characteristics are shown in Table 1. Each patient’s CT examination was performed on the same day or later than the MRI; the intervals between CT and MRI were 0–52 days (median 6 days). The CTDIvol values for the spectral CT were 8.0–24.1 mGy (median 11.1 mGy) and the DLP data were 393.5–1842.0 mGy･cm (median 714.2 mGy･cm).

**Fig. 3:**
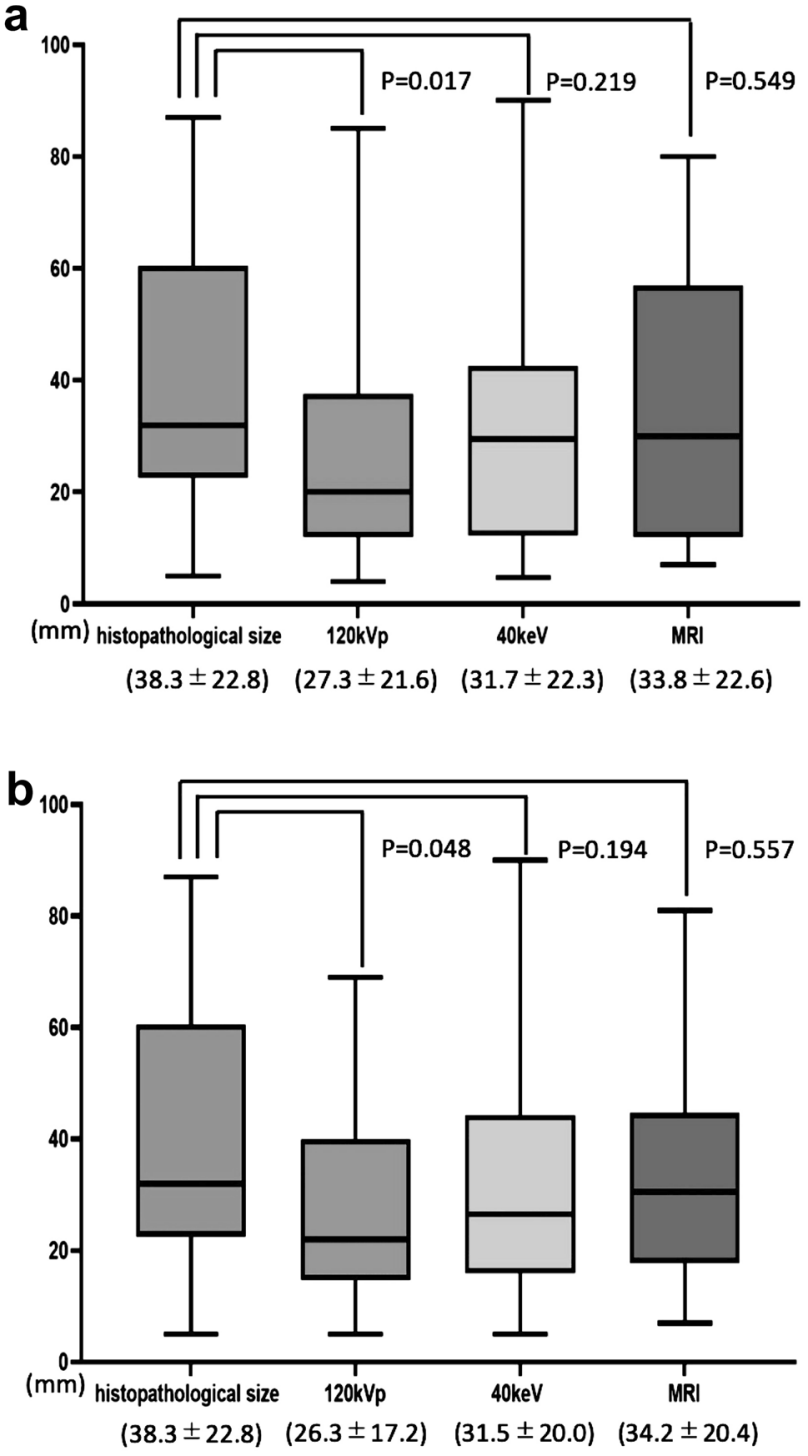
Comparison of the measured lesion sizes and the sizes revealed by histopathology. The measurements on the conventional 120-kVp CT were significantly smaller than histopathological sizes for both of Reader 1 (**a**) and Reader 2 (**b**)

**Fig. 4:**
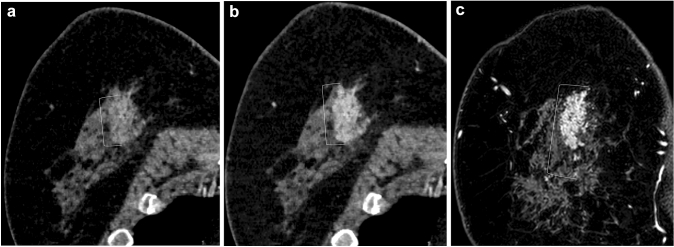
A 49-year-old female with ductal carcinoma in situ (histopathological size 60 mm) (Group 2). On coronal images of conventional 120-kVp CT (**a**), 40-keV VMI (**b**), and MRI (**c**), Reader 1 measured the lesion size as 37 mm, 44 mm, and 57 mm, and Reader 2 measured the size as 39 mm, 44 mm, 53 mm, respectively. Conventional 120-kVpCT underestimated the lesion size compared to MRI and 40-keV VMI

**Fig. 5:**
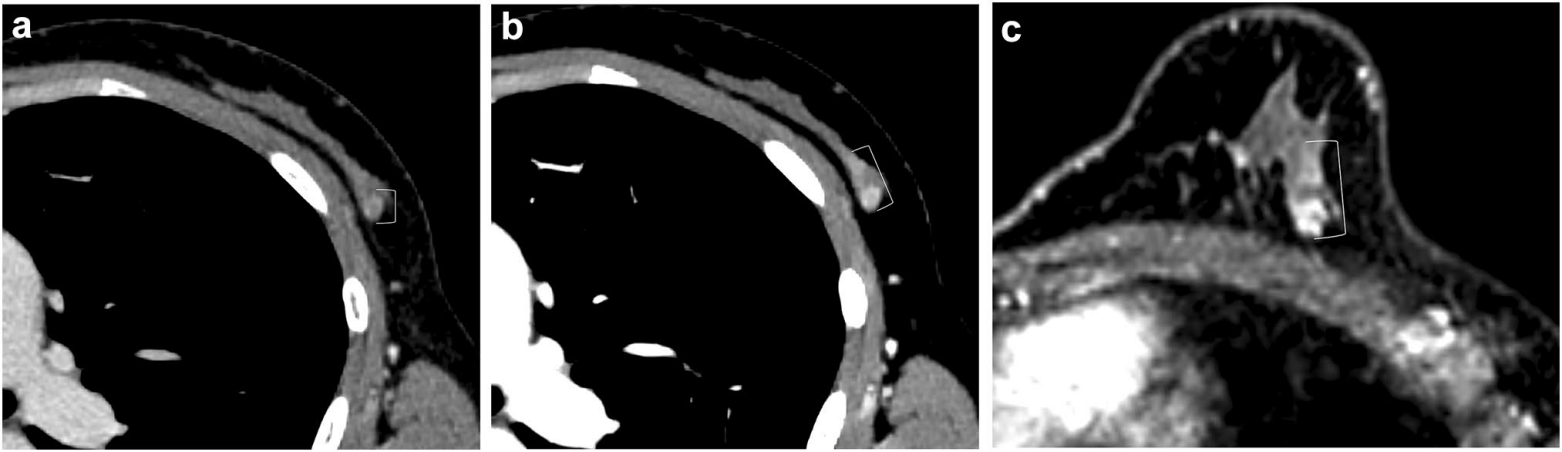
A 46-year-old female with microinvasive carcinoma (histopathological size 18 mm) (Group 2). On axial images of conventional 120-kVp CT (**a**), 40-keV VMI (**b**), and MRI (**c**), Reader 1 measured the lesion size as 6 mm, 7 mm, and 9 mm, and Reader 2 measured the size as 7 mm, 14 mm, and 18 mm, respectively. The conventional 120-kVp CT underestimated the lesion size compared to MRI and 40-keV VMI. The measurement of 40-keV VMI was much closer to that of MRI compared to conventional 120-kVp CT

Both of the readers significantly underestimated the lesion size on conventional 120-kVp CT images compared to the histopathological size (Reader 1, 27.3 ± 21.6 mm vs. 38.3 ± 22.8 mm, *p* = 0.017; Reader 2, 26.3 ± 17.2 mm vs. 38.3 ± 22.8 mm, *p* = 0.048). Although the lesion sizes documented by MRI (Reader 1, 33.8 ± 22.6 mm; Reader 2, 34.2 ± 20.4 mm) and those obtained with 40-keV VMI (Reader 1, 31.7 ± 22.3 mm; Reader 2, 31.5 ± 20.0 mm) were also smaller than those observed on the histopathological evaluation (38.3 ± 22.8 mm), the differences were not significant (Reader 1, *p* = 0.549, 0.219; Reader 2, *p* = 0.557, *p* = 0.194) (Figs. 3, 4, 5).

**Fig. 6:**
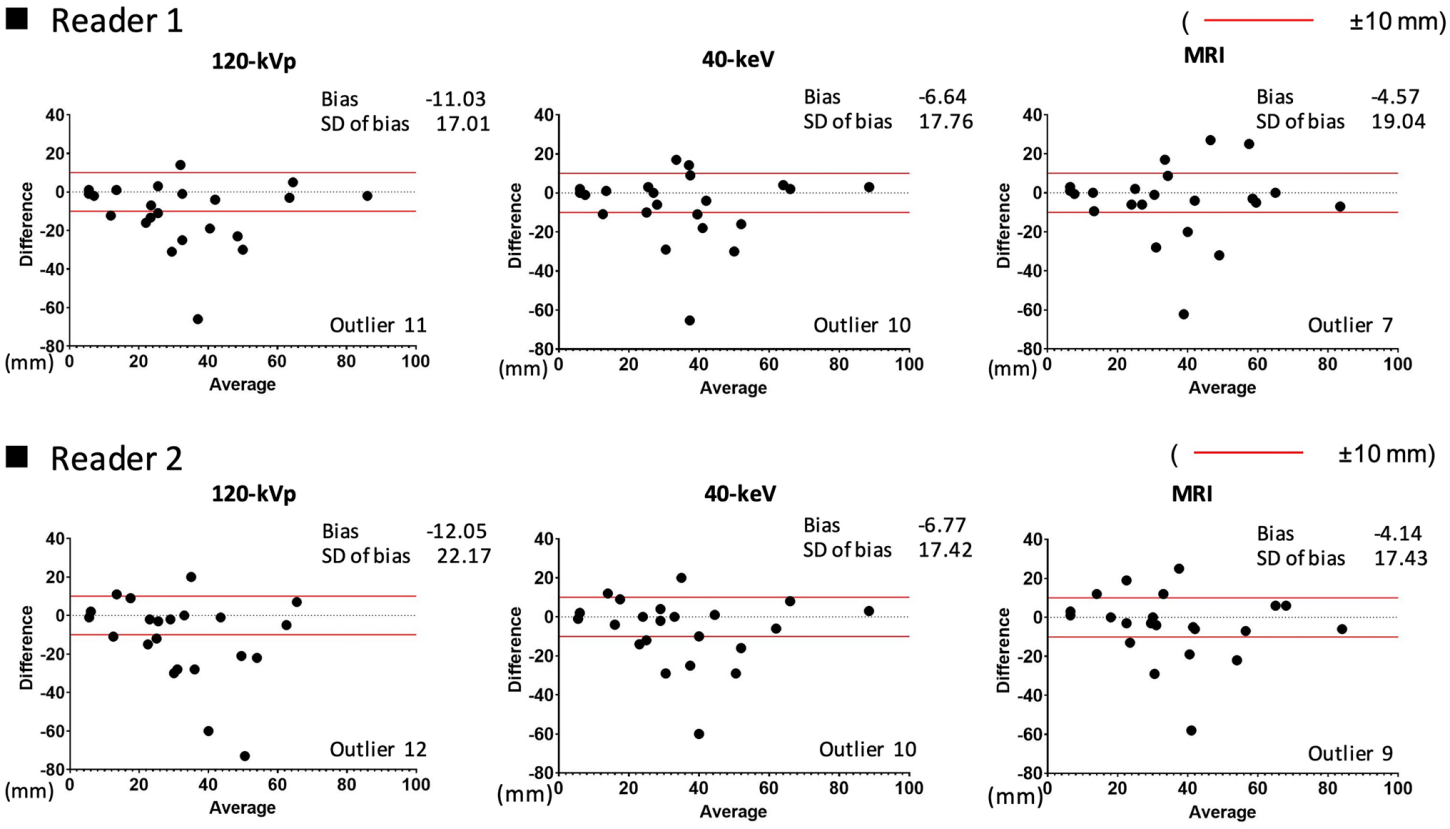
The agreement between the histopathological lesion size and measurements on each modality. Bland–Altman plots show the degree of agreement between conventional 120-kVp CT and histopathology, 40-keV VMI and histopathology, and MRI and histopathology for each reader. When compared with the histopathological size, MRI showed the highest number of lesions within a 10-mm error range, followed by 40-keV VMIs and conventional 120-kVp CT images

**Fig. 7:**
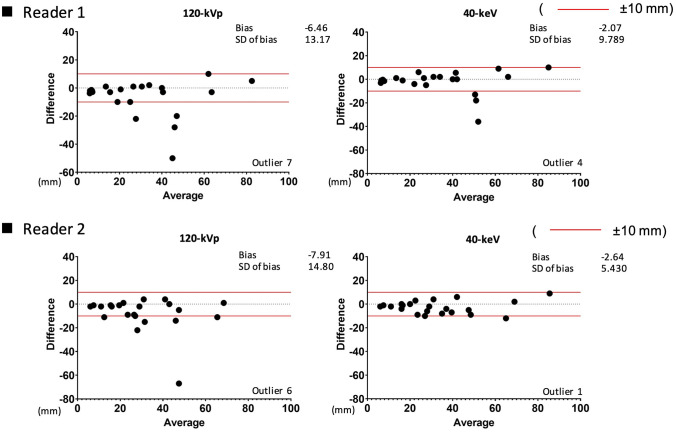
The agreement between the measurements on CT and MRI. Bland–Altman plots show the degree of agreement between conventional 120-kVp CT and MRI, and between 40-keV VMI and MRI for each reader. When compared with MRI, 40-keV VMI demonstrated measurement within a 10-mm error range in more lesions than conventional 120-kVp CT images

When compared with the histopathological size, the MRI results showed the highest number of lesions within a 10-mm error range (Reader 1, *n* = 15 cases; Reader 2, *n* = 13), followed by 40-keV VMI (both Readers, *n* = 12) and conventional 120-kVp CT images (Reader 1, *n* = 11 cases; Reader 2, *n* = 10) (Fig. 6). When the comparison was made with measurements on MRI, 40-keV VMI demonstrated measurement within a 10-mm error range in more lesions compared to the conventional 120-kVp CT images (Reader 1, 18 cases vs. 15 cases; Reader 2, 21 cases vs. 16 cases) (Fig. 7). The interobserver reliability of 40-keV VMI was excellent [ICC (2,1) = 0.96] and that of MRI was good [ICC (2,1) = 0.83], whereas that of conventional 120-kVp CT was moderate [ICC (2,1) = 0.66].

## Discussion

MRI has been reported to detect intramammary spread of breast cancer with higher sensitivity due to its excellent contrast, whereas CT has been described as less sensitive in detecting tumor progression due to its low contrast [5–7]. We speculated that low-energy-level VMI could increase the contrast enhancement of breast cancer and improve the detectability of intramammary spread. We thus evaluated 40-keV VMI, the lowest-energy-level image that our equipment was able to reconstruct. In our present investigation, the CNR values obtained with CT were improved by using spectral CT to generate 40-keV VMI, resulting in sensitivity for intramammary extension that was comparable to that of MRI. The results of our analysis of Group 1 demonstrated that 40-keV VMI provided improvement in the CNRs compared to the conventional 120-kVp CT images. We speculate that in Group 2, the improved CNRs contributed to the improved detection of intramammary spread. Inoue et al. and Okada et al. evaluated VMIs at various energy levels and observed that the CNR and tumor visibility on 40-keV VMI were higher than those on conventional 120-kVp CT images [9, 10], which is consistent with our present findings. Another study detected no significant difference between lesion sizes observed on dual-energy CT and the sizes obtained by pathological analysis [8]; however, most of that study’s subjects had invasive carcinoma, which is assumed to be easier to detect. Our present results demonstrated that the use of 40-keV VMI could detect even the spread of noninvasive or microinvasive carcinomas.

In the comparison with MRI, 40-keV VMI produced measurements within a 10-mm error range in most lesions, suggesting a diagnostic ability equivalent to that of MRI. The evaluation of intramammary spread and systemic staging could therefore be performed simultaneously using VMI. Indeed, our results suggest that VMI could be used for a comprehensive evaluation of breast cancer without the use of MRI.

Another advantage of CT for preoperative evaluation is the consistent patient positions. Breast MRI is commonly performed with the patient in the prone position with the breasts pendant into specialized coil arrays in order to minimize breast motion due to respiration, reduce the potential interference with the beating heart, and improve the coil coupling [11]. Depending on the part of the breast, a tumor’s location can change remarkably when the patient’s position changes from prone to supine [12]. Thus, the location of the lesion in MRI performed in the prone position is sometimes differs from that in the surgery performed with the patient in the supine position. CT images can be obtained with the supine position, which is compatible with the surgical situation.

MRI has the advantage of providing a dynamic contrast-enhanced study. The CT that was performed for the present patients was a one-phase examination, and we thus speculate that the CT examinations were more influenced by background parenchymal enhancement (BPE) than MRI. In some patients with marked BPE, the CNRs of the lesions were actually reduced on 40-keV VMI. In such cases, other phases of the dynamic study were obtained on MRI. The usefulness of ultrafast dynamic contrast-enhanced MRI has also been reported [13]; however, multiphase imaging on CT is not always recommended due to the radiation exposure.

There are several dual-energy CT techniques including dual X-ray sources, rapid kV switching, and the use of a dual-layer detector, the latter of which was used for the present patients. Dual-layer detector CT allows the differentiation of the two datasets at the detector level, and it therefore enables the acquisition of dual-energy information without increasing the radiation dose compared to a conventional single-layer detector CT. Dual-layer detector CT can thus provide both conventional CT and VMI without increasing the radiation dose, and conventional CT can be referenced as needed when the CNR obtained by VMI is low. Additionally, other organs can be observed under conventional CT.

There are some limitations to our study. First, its design was retrospective and the number of subjects was small. Prospective studies with larger populations are required to confirm the efficacy of VMI for evaluating the intraductal spread of breast cancer. Second, the measurement direction on each modality may not have matched in all cases, although the readers sought out the maximum diameters of the lesions. There may also be a difference in size between CT and MRI due to the difference in the patient’s position. Third, the patients’ CT and MRI were not always performed on the same day. The interval was < 2 weeks in most cases, but there were a few cases in which the interval was nearly 50 days. However, we surmise that there was no significant change in diameter, because these patients had DCIS or microinvasive carcinoma. In addition, the degree of BPE was not evaluated. BPE is known to be associated with the menstrual cycle, and marked BPE can decrease the diagnostic performance [14]. When a patient’s CT and MRI were not performed on the same day, the effects of BPE may have differed due to differences in the patient’s menstrual cycles.

In conclusion, our retrospective analyses demonstrated that VMI improved the capability of CT for the evaluation of breast cancer intraductal spread, and its use provided diagnostic performance that is closer to that of MRI. VMI, which can provide simultaneous assessments of intramammary spread and whole-body cancer staging, is thus especially useful for the preoperative evaluation of breast cancer.

**Table 1:** Patients’ characteristics

	Group 1 (*n* = 24)	Group 2 (*n* = 22)
Age (years)	range 34–74; median 65	range 37–82; median 56
Histopathological diagnosis		
Ductal carcinoma in situ	1	16
Microinvasive carcinoma	2	6
Invasive ductal carcinoma	21	0
Invasive size (mm)	range 2–70; median 18	
Subtype		
Luminal	15	
Luminal-HER2	3	
HER2	2	
Triple-negative	1	
Stage		
pN0/pN1/pN2/pN3	19/3/2/0	22/0/0/0
M0/M1	24/0	22/0

Note. Except where indicated, data are numbers of patients. HER2 = human epidermal growth factor receptor 2
